# Proton Reirradiation for High-Risk Recurrent or New Primary Breast Cancer

**DOI:** 10.3390/cancers15245722

**Published:** 2023-12-06

**Authors:** Molly A. Chakraborty, Atif J. Khan, Oren Cahlon, Amy J. Xu, Lior Z. Braunstein, Simon N. Powell, J. Isabelle Choi

**Affiliations:** 1 Rutgers New Jersey Medical School, Newark, NJ 07103, USA; 2 Department of Radiation Oncology, Memorial Sloan Kettering Cancer Center, New York, NY 10065, USA; 3 Department of Radiation Oncology, New York University, New York, NY 10016, USA; 4 New York Proton Center, New York, NY 10035, USA

**Keywords:** breast cancer, radiation therapy, reirradiation, proton therapy

## Abstract

**Simple Summary:**

Photon reirradiation (reRT) has often been avoided in patients with breast cancer recurrences or new primary breast cancer following prior radiation to the thoracic region due to concerns for severe toxicities from high cumulative dose delivery. However, some patients in this setting may benefit from a repeat course of definitive-intent reRT to improve locoregional control and potentially enhance survival outcomes. The aim of this review article is to highlight the potential of proton therapy as a method to more safely deliver curative, tumoricidal doses and more comprehensive reRT for breast cancer. Herein, we discuss which patient populations might benefit the most from proton breast reRT, review the available literature in this area, discuss reRT treatment planning considerations, and highlight questions in this space in need of further exploration.

**Abstract:**

Radiotherapy is an integral component of multidisciplinary breast cancer care. Given how commonly radiotherapy is used in the treatment of breast cancer, many patients with recurrences have received previous radiotherapy. Patients with new primary breast cancer may also have received previous radiotherapy to the thoracic region. Curative doses and comprehensive field photon reirradiation (reRT) have often been avoided in these patients due to concerns for severe toxicities to organs-at-risk (OARs), such as the heart, lungs, brachial plexus, and soft tissue. However, many patients may benefit from definitive-intent reRT, such as patients with high-risk disease features such as lymph node involvement and dermal/epidermal invasion. Proton therapy is a potentially advantageous treatment option for delivery of reRT due to its lack of exit dose and greater conformality that allow for enhanced non-target tissue sparing of previously irradiated tissues. In this review, we discuss the clinical applications of proton therapy for patients with breast cancer requiring reRT, the currently available literature and how it compares to historical photon reRT outcomes, treatment planning considerations, and questions in this area warranting further study. Given the dosimetric advantages of protons and the data reported to date, proton therapy is a promising option for patients who would benefit from the added locoregional disease control provided by reRT for recurrent or new primary breast cancer.

## 1. Introduction

Breast cancer remains the most common non-cutaneous malignancy in women, with an estimated 2.26 million cases diagnosed worldwide annually [1]. Although locoregional recurrence rates are low in patients receiving modern multidisciplinary treatment for non-metastatic breast cancer, the risk for recurrence or the development of a new primary breast cancer can persist for many years after definitive treatment [2,3,4]. While the relative risk of breast cancer recurrence is overall low, the number of patients represented is a significant population given the high overall incidence of the disease. Radiotherapy (RT) is commonly used in the adjuvant treatment of localized breast cancer to reduce recurrence risk and improve survival; thus, many patients with breast cancer recurrences have previously received RT [5,6]. Additionally, some patients, upon their initial presentation with breast cancer, may have had prior RT to the thoracic region for diseases such as Hodgkin lymphoma [7].

Historically, there has been a lack of consensus on the optimal approach to the treatment of breast cancer patients who have received prior RT to the breast and thoracic regions. Salvage mastectomy in the setting of prior breast-conserving therapy, or, more recently, repeat breast-conserving surgery followed by partial breast irradiation (PBI) or brachytherapy, have been commonly used approaches to date [8,9,10,11,12,13,14]. In the post-mastectomy or high-risk recurrent settings in which repeat breast conservation is not a viable option, photon reirradiation (reRT) that is more comprehensive and of accepted tumoricidal dose has often been avoided due to concerns for high cumulative doses to organs-at-risk (OARs) such as the skin, soft tissue, heart, lungs, ribs and brachial plexus, which could potentially lead to unacceptable rates of severe toxicities [15,16]. However, higher-dose and more comprehensive adjuvant reRT could be beneficial for disease control, especially for patients whose recurrences have adverse disease features such as muscle invasion, dermal/epidermal invasion, lymphovascular invasion, close/positive surgical margins, or lymph node involvement, as these patients are still at considerable risk for recurrences after surgery [6]. Additionally, higher dose adjuvant reRT may be necessary as recurrent tumors could represent a clonal population with more aggressive underlying biology or with a radioresistant phenotype [17,18].

Proton beam therapy (PBT) is a potentially advantageous treatment option that may allow for higher doses and more comprehensive reRT without significantly increasing the dose to OARs and associated toxicities due to its greater ability to spare normal tissue in the treatment of breast cancer [19,20,21,22,23]. Herein, we review the existing literature and discuss the potential clinical applications of PBT for breast cancer patients who have received prior RT to the breast and thoracic regions.

## 2. Body

### 2.1. Toxicities of Reirradiation

Even with the most advanced photon therapy technology for breast cancer currently available, adjacent organs are exposed to radiation and are thus at risk for toxicities [24]. Radiation to these adjacent OARs can lead to effects such as radiation dermatitis, fibrosis, rib fractures, chronic chest wall/breast pain, shoulder stiffness, brachial plexopathy, radiation pneumonitis, and, perhaps most concerning, cardiac disease, especially in patients being treated for left-sided breast cancers. Additionally, patients receiving photon RT for breast cancer may be at increased risk for developing contralateral breast cancers or other subsequent malignancies such as sarcomas [25,26].

A population-based case–control study of patients who received RT for breast cancer by Darby et al. found that increases in mean radiation dose to the heart linearly correlate with a higher risk of major coronary events: for every additional gray (Gy) of radiation exposure, the risk of major coronary events increased by 7.4%, with no dose threshold [27]. A study performed by van der Bogaard et al. found that there was a 16.5% increase in the risk of developing major coronary events per additional Gy of radiation exposure to the heart during breast RT [28]. Additionally, a study performed by Taylor et al. found that increased radiation dose to coronary artery segments also correlates with a higher risk for cardiac disease [29]. Another study found a correlation between radiation dose to the lungs and risk of secondary pulmonary malignancy [30]. The results of these studies suggest that increased radiation dose, such as that from breast/chest wall reRT, may lead to significantly increased risks of cardiac and pulmonary toxicities, as well as toxicities related to other adjacent OARs.

Indeed, full-dose photon reRT has largely been avoided due to concerns of higher doses to OARs, which can lead to severe toxicities that impact quality of life and survival [31]. Additionally, a study investigating photon reRT of breast cancer patients found that higher reRT dose is associated with an increased likelihood of developing late grade 3 toxicities [16].

### 2.2. Patient Selection and Rationale for Proton Therapy

Salvage breast-conserving surgery followed by PBI has been shown to be feasible in small, low-risk recurrences, but this approach is not optimal in the setting of bulky or high-risk recurrences [9]. Patients with adverse clinicopathologic disease features, such as large tumor size, dermal/epidermal invasion, muscle/chest wall involvement, close or positive surgical margins, lymphovascular invasion, and lymph node involvement, benefit from the added locoregional control afforded by salvage RT given their substantial risk for future recurrences to the breast, chest wall, and regional lymph node basins after surgical resection alone [6]. Thus, patients with high-risk recurrences or new primary breast cancers undergoing salvage breast-conserving surgery or mastectomy may also benefit from higher reRT doses and more comprehensive adjuvant reRT. In patients who were treated in the primary setting with mastectomy and then received maximal surgical resection for chest wall recurrence, a comprehensive target coverage approach to the chest wall and regional lymph nodes is also indicated, as these tumors often possess one or more of the adverse features listed above. Patients who present with a nodal recurrence are also at risk for recurrence to the nodal basins, which also lends concern for tumor seeding of the chest wall due to the potential for aberrant lymphatic drainage after prior surgery and RT. A study found that patients with internal mammary node (IMN) recurrences benefit from radiation but are very difficult to manage, given the proximity of the IMNs to the heart, suggesting that reRT may be particularly beneficial for patients with this high-risk feature [32]. Additionally, some patients may be medically inoperable, in which case reRT would be the only option for local disease control. 

In such cases, PBT would be beneficial compared to photons as they result in reduced additional radiation dose to non-target tissues and surrounding OARs, minimizing the incidence and severity of toxicities following reRT. In comparison to photons, protons have increased atomic mass and carry a positive electrical charge, which results in differential physical properties that confer advantages in dose conformality, homogeneity, and normal tissue sparing. As protons enter the body, they deposit dose slowly, resulting in a lower entrance dose compared to photons. However, the dose delivered to the skin and superficial targets with pencil beam scanning proton therapy, which is of particular importance in the setting of chest wall recurrences often involving the dermis and epidermis and requiring full prescription dose delivery to the skin, can be adequately modulated to reach the target prescription dose without the use of bolus [33]. In one dosimetric study, the mean skin surface dose was 72.2%, 70.9%, 98.1%, and 108.4% with the use of prone photons without bolus, supine photons without bolus, protons without bolus, and photons with bolus, respectively (*p* < 0.001) [34]. Near the end of their range, protons deposit much of their energy very rapidly over a small volume, resulting in a sharp dose falloff known as the Bragg peak. Beyond the Bragg peak, no energy is deposited, resulting in no exit dose [35,36]. Thus, while in-field toxicities cannot be avoided by protons, protons do allow for less dose to be delivered to OARs proximal and distal to the target volume during breast RT compared to external beam photon techniques, potentially reducing associated toxicities and providing properties that are particularly desirable for reRT [19,20,21,35,36]. Additionally, while brachytherapy is more commonly employed for breast reRT in some areas of the world, PBT may reduce the dose heterogeneity associated with this approach, and it allows for more comprehensive target volume coverage compared with this modality [37].

**Figure 1:**
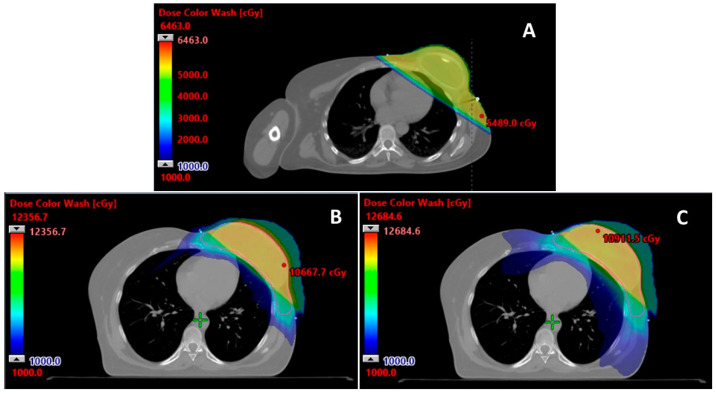
Representative axial radiation plan images of a female patient with a primary diagnosis of cT2N0M0 left breast cancer status post neoadjuvant chemotherapy followed by left total mastectomy and axillary lymph node dissection, ypT2N2aM0, followed by comprehensive post-mastectomy radiotherapy (RT) to the left chest wall (CW) and regional lymph node (LN) basins to 50 Gy in 25 fractions, then 7.4 years later with left CW recurrence s/p excision with high-risk features (skeletal muscle and fibroadipose tissue invasion, positive surgical margins, lymphovascular invasion), who received reirradiation to the left CW and regional LN basins. (**A**) Initial photon radiation plan, (**B**) cumulative initial photon and reirradiation proton plan, and (**C**) cumulative initial photon and reirradiation volumetric-modulated arc therapy (VMAT) plan. Colorwash: low dose (blue) = 1000 cGy to DMax (red).

Indeed, many studies have demonstrated that PBT results in a large reduction in cardiac and coronary artery radiation dose when compared to photon techniques, including three-dimensional conformal radiation therapy (3DCRT), intensity-modulated radiation therapy (IMRT), and volumetric-modulated arc therapy (VMAT) [38,39,40,41,42,43,44]. Multiple studies have also demonstrated that PBT results in significantly reduced doses to cardiac tissues and substructures compared to photon techniques combined with deep inspiration breath hold (DIBH) [39,40,41]. Thus, reRT using PBT can avoid the risk of cardiac toxicities associated with repeat RT exposure to the heart in a non-primary RT course (Figure 1). Similarly, studies have demonstrated that PBT results in reduced radiation dose to the lungs compared to photon RT. Reduction in high dose (≥20 Gy) delivery to the lungs achievable by PBT may allow patients to receive reRT without significantly increased risk of pulmonary toxicities, including pneumonitis and secondary pulmonary malignancies [45,46,47,48]. Sparing of other OARs may allow for patients to not be at significantly increased risk for other associated toxicities as well.

This may be of particular importance for patients who are at a baseline greater risk for developing severe toxicities, such as patients with bilateral recurrences and those with pre-existing cardiac disease risk factors [27,49]. A study also found that patients with a short interval between initial and repeat RT courses are more likely to experience severe toxicities, suggesting that patients with earlier relapses may also particularly benefit from PBT [15]. 

In patients who receive a “high tangent field” or comprehensive regional nodal irradiation for primary breast cancer presentation who then present with high-risk breast cancer recurrence requiring repeat treatment of the regional nodal basins at risk, the brachial plexus is an OAR of concern, as two curative courses of breast RT to this structure would far exceed its acceptable cumulative dose limit. Brachial plexus injury can result in significant detrimental clinical manifestations, including arm loss of movement, weakness, paresthesia, and pain. Intensity-modulated proton therapy (IMPT) can reduce the dose delivered to the brachial plexus in the setting of reRT while maintaining dose coverage to the surrounding target tissue to a greater degree than that achievable with advanced conformal photon techniques such as VMAT [50]. Therefore, for patients requiring a repeat course of radiation to areas that overlap with a previously irradiated brachial plexus, such as in breast cancer patients who require repeat regional nodal irradiation, proton therapy is indicated.

Potential exceptions to the inclusion of all surrounding lymph node basins and/or breast or chest wall reRT for high-risk breast cancer recurrence include those patients with very short intervals to recurrent disease presentation in whom there is a reasonably high likelihood that the recurrence represents under-treated primary disease (i.e., short interval <1 year IMN recurrence in a patient who was treated comprehensively with wide tangent fields that did not adequately cover the IMN chain to achieve acceptable cardiac sparing). In these rare situations, consideration could be given to the treatment of the newly involved nodal basin only, given the relatively low index of suspicion of new tumor seeding elsewhere.

### 2.3. Studies Investigating Proton ReRT for Breast Cancer

A study from the prospective, multicenter Proton Collaborative Group (PCG) registry included 50 patients who received PBT reRT for breast cancer from 2011 to 2016. A total of 24% of patients received pencil beam scanning (PBS)-PBT, 52% received uniform scanning PBT, and 24% had unspecified PBT type. The median prior RT dose was 60 Gy, the reRT dose was 55.1 Gy(RBE), and the total dose was 110.6 Gy(RBE). The reRT plans included regional lymph nodes for 84% of the patients and IMNs for 66% of the patients. At a 12.7-month median follow-up, three patients developed a local recurrence, four patients had metastatic recurrences, and six patients died due to breast cancer (12-month locoregional free survival (LRFS) 93%, overall survival (OS) 97%). Acute grade 3 toxicities (within 180 days of the start of reRT) were experienced by 10% of patients, whereas late grade 3 toxicities were experienced by 8% of patients. Of note, grade 3+ effects were only experienced by patients receiving radiation to IMNs. A body mass index (BMI) of greater than 30 and bilateral recurrence were also associated with increased risks of grade 3 toxicities. Patients with gross disease at the time of PBT reRT trended towards decreased 12-month LRFS and OS (*p* = 0.06 and *p* = 0.14, respectively). The study concluded that the toxicities were acceptable and that the LRFS and OS were similar to previous reRT studies despite the fact that many of the patients in the study had gross disease at the time of reRT (26% of the patients did not undergo surgery before reRT) [51].

A small retrospective study from Washington University investigated 16 patients who underwent passive scattering PBT reRT for breast cancer between 2013 and 2018, with a median follow-up of 18.7 months. The median prior RT dose was 50 Gy, followed by a median boost dose of 10 Gy in patients who required a boost. The median reRT dose was 50.4 CGyE in 28 fractions (1.8 CGyE per fraction). There were no local or regional recurrences, but there was one metastatic recurrence leading to the death of that patient (18-month local control 100%, distant control 93.3%, overall survival 88.9%). Five patients (31.2%) experienced acute (within eight weeks of the start of treatment) grade 3–4 skin toxicities, four patients (25%) developed acute chest wall infections, three patients (18.8%) developed late grade 3–4 fibrosis, two patients (12.5%) experienced radiation pneumonitis, and no patient experienced cardiac toxicities. The study concluded that the toxicities were acceptable overall, but higher rates of grade 3+ skin effects were observed in comparison to previous studies investigating photon reRT, such as the large Wahl et al. study published in 2008, possibly due to the concurrent use of hyperthermia as a radiosensitizer [52,53].

Investigators at the Mayo Clinic studied 72 patients who underwent reRT for breast cancer between 1999 and 2019, 52 (72%) of whom received photons with or without electrons and 20 (28%) of whom received IMPT. The median prior RT dose, reRT dose, and total dose were 50 Gy, 45 Gy, and 103.54 Gy_2_, respectively. A total of 47 patients (65%) received conventionally fractionated treatment, 21 (29%) received hypofractionated treatment, and 4 (6%) received hyperfractionated treatment. Dosimetric advantages were observed in PBT plans, but at a median follow-up of 22 months, there were no differences observed in toxicities in patients undergoing PBT in comparison to patients undergoing photon reRT. Overall, 13% of patients experienced grade 3 adverse effects. Fifty-two (44%) patients had gross disease at the time of reRT, and twenty-eight (39%) had treatment plans that were palliative in intent. When considering all patients, the rates of two-year LRFS and OS were 74.6% and 65.6%, respectively. When only considering patients who were treated with curative intent, the LRFS was 93.1%, and the OS was 76.8%. Distant control was 59% among patients who were treated with curative intent. A total of 13%of patients experienced grade 3 toxicities, of which 3% were late effects. Of note, late grade 3 events occurred only in patients receiving photons. The study concluded that the toxicities were acceptable, with promising local control rates for patients who were treated with curative intent [15].

At the University of Pennsylvania, 27 patients treated with PBT reRT for breast cancer between 2012 and 2019 were retrospectively evaluated. A total of 29% of these patients received double scattering PBT, 66.7% received PBS-PBT, and 3.7% received combined double scattering and PBS-PBT. The median follow-up was 16.6 months, and patients were treated to a median reRT dose of 51 Gy, which was most commonly delivered in twice-daily fractions of 1.5 Gy. ReRT dose in EQD2 was 45.6 to 45.9 in 19 patients (70.4%), 37.8 in 1 patient (3.7%), 50.0–60.0 in 6 patients (22.22%), and 66.0 in 1 patient (3.7%). There was one patient (3.7%) who had a local recurrence, eleven patients (40.6%) had distant recurrences, six (22.2%) died due to breast cancer, and one died due to an unknown cause (12-month LRFS 78.5%, recurrence-free survival 62.4%, OS 78.5%). Two patients (7.4%) experienced grade 3 acute radiation dermatitis, two patients (7.4%) experienced grade 3 acute breast pain, one patient (3.7%) experienced grade 3 late dermatitis, and one patient (3.7%) developed late grade 3 breast pain. One patient (3.7%) who received concurrent capecitabine experienced grade 4 late dermatitis [54]. Of note, there were six patients (22.2%) who developed grade 2 rib fractures, which the authors postulated might be due to the probable higher biological effectiveness of protons at the end of their range, especially in light of another study finding higher rates of rib fracture following PBT compared to photon radiotherapy for breast cancer [54,55]. The study concluded that locoregional control was excellent following the hyperfractionated PBT reRT regimen, and toxicities were acceptable [54].

A retrospective study from investigators at Memorial Sloan Kettering Cancer Center included 46 patients treated with PBT reRT for breast cancer between 2012 and 2020, 70% of whom received uniform scanning PBT and 30% of whom received PBS-PBT. The median follow-up was 21 months, with a median initial RT dose, reRT dose, and total dose of 60 Gy (58.93 Gy_2_), 50.4 Gy(RBE) (50 Gy_2_), and 110 Gy(RBE) (108.9 Gy_2_), respectively. Forty-four patients (95.7%) were treated with conventional fractionation for reRT, except for two (4.3%) who received twice daily fractions of 1.5 Gy each for PBI. There were no local or regional recurrences, but 17% had distant recurrences, and 7% passed away. The estimated 2- and 3-year distant metastasis free survival (DMFS) were 92% and 60%, respectively, and the estimated 2- and 3-year OS were 93.6% and 88.1%, respectively. Nearly one-third (30.4%) of patients experienced acute (within three months of PBT) grade 3 events, and 8.7% experienced late grade 3 effects. Of note, late toxicities were limited to in-field structures, which cannot be spared by either photons or protons. The study concluded that locoregional control was excellent, with few high-grade toxicities reported [56].

A retrospective study from Rutgers Cancer Institute of New Jersey included 15 patients who underwent reRT for breast cancer between 2015 and 2020, 80% of whom received PBT. The median follow-up was 14 months, with a median prior breast/chest wall (CW) dose and reRT dose of 50 Gy and 45 Gy(RBE), respectively. Over one-quarter (27%) of patients experienced locoregional recurrence, and 33% developed distant metastasis. Only 13% of patients experienced any grade 3 toxicity. The study concluded that reRT, which in most cases was delivered using PBT, allowed for low rates of locoregional recurrence with low rates of severe toxicities [57].

Of note, two of the studies concluded that distant recurrence is a major mode of disease control failure, suggesting that systemic therapies for local recurrences need to be improved and ideally should be a component of treatment for recurrences [54,57]. Interestingly, the Choi et al. study found that higher BMI may have a protective effect against acute grade 2+ toxicities, in contrast to the Thorpe et al. study, which found that increased BMI was associated with increased risk for severe toxicities [51,56]. 

One of the largest studies on photon reRT was published by Wahl et al. in 2008. The study included 81 patients with median prior RT and reRT doses of 60 Gy and 48 Gy, respectively. Late grade 3+ toxicities were observed in 5.7% of patients. The overall one-year LRFS was 66%, but for patients who did not have gross disease at the time of reRT, the LRFS was 100%. The one-year OS was 64% [53]. Overall, the outcomes and toxicities reported in this study appeared to be similar to the reported outcomes of PBT reRT, but many of the proton studies did find better one-year LRFS and OS rates. Indeed, a recent consensus statement from the Particle Therapy Cooperative Group (PTCOG) Breast Cancer Subcommittee also concluded that outcomes from PBT reRT appear to be similar to outcomes from photon reRT studies, but there is as yet limited long-term follow-up on patients who have received PBT reRT [37]. Continued follow-up of these patients is necessary to understand if the dosimetric advantages achieved by PBT for breast reRT correlate with improved long-term toxicity outcomes, such as decreased rates of cardiac disease or brachial plexopathy. While in the primary breast cancer treatment setting, optimal preservation of cosmesis is a significant consideration in the overall plan of care, in the recurrent setting because of the need for multiple surgical and/or radiotherapeutic interventions in which the skin and soft tissue are repeatedly subject to manipulation, compromise of this outcome may be accepted in an effort to maximize the likelihood for successful cancer salvage. Table 1 summarizes the key characteristics of each of the described studies.

### 2.4. RBE/LET Considerations

The relative biological effectiveness (RBE) for PBT is the ratio of photon dose needed to achieve the same biological effect as an equivalent nominal proton dose. Historically, an RBE value of 1.1 has been universally assigned for proton therapy. However, recent evidence suggests that the RBE of protons varies from that value in different situations and is not a constant. For example, the RBE value will be higher at the end of the proton’s range, as the linear energy transfer (LET) of protons is highest in this region [58,59,60]. Thus, since the ribs and intercostal spaces are usually just distal to the edge of the target region in PBT planning for breast cancer, more biological doses than expected might be delivered to these structures [37]. Additionally, technical variations in proton delivery can also influence biological effects. In comparison to passively scattered techniques, scanning techniques can lead to increased LET outside of the clinical target volume (CTV) and greater RBE variability [61,62]. RBE variability may lead to biologic hotspots and increased dose to structures just distal to the edge of the target region, such as the ribs and intercostal spaces, leading to toxicities such as rib fractures [54,55,63,64,65]. Indeed, the LaRiviere et al. PBT reRT study, along with studies by Jimenez et al. and Wang et al., found increased rates of rib fractures compared to historical photon rates, supporting the idea that protons have a higher RBE value at the end of their range. The Jimenez et al. study utilized passive scattering and PBS PBT, patients in the LaRiviere et al. study received proton double scattering PBT or PBS PBT, and patients in the Wang et al. study received passively scattered PBT, PBS PBT, or accelerated partial breast irradiation (APBI) PBT [54,55,65]. Of note, a recently published study of patients receiving either passively scattered or PBS PBT found that the incidence of rib fractures was similar to that of historical photon rates, contrary to the findings of the other studies [66].

There is currently a lack of consensus on how to optimally account for RBE variability in PBT treatment of breast cancer. In a study from the Mayo Clinic, several adjustments were made to account for RBE value variability without significantly changing the CTV coverage in breast cancer treatment: (1) implementing a brachial plexus dose constraint, (2) using at least two to three fields to prevent potential biologic hotspots and range extension, and (3) accepting 90–95% of the prescription dose in the most posterior millimeters of the target range [63]. Methods such as using LET-weighted doses may be able to be utilized in the future to reduce RBE variability [67].

### 2.5. Treatment Planning Approach and Considerations

CTV delineation is essential in PBT treatment planning due to the sharp dose falloff at the end of the proton beam range. The effect of range uncertainty must also be considered when planning for PBT. When planning for breast cancer treatments, the composite distal margins used to account for this uncertainty are usually approximately 2–3 mm in order to avoid excess doses to OARs such as the heart and lungs [37]. 

Additionally, due to the sharp distal dose fall off of the proton beam, along with the en-face orientation of proton beams, PBT is more sensitive to variations in set-up [24,61,68]. Changes in the size of the target, such as contraction due to the resolution of a seroma or expansion due to edema, can lead to undesired dose to structures distal to the planned range or under coverage of distal parts of the target range, respectively [24,37,61]. Surface imaging and interval verification scans have been utilized to identify interfraction and intrafraction variations in anatomy [69,70,71]. Adaptive re-planning can be used for patients with significant anatomy variations, but this tends to be required for only a small minority of patients [24,37,72].

Patients with breast implants or tissue expanders require extra consideration because the stopping power of protons is dependent on the material they are traveling through, and implants and expanders can consist of material that has a very different stopping power than normal breast tissue. Stopping power is required to calculate the range of protons, so failing to consider changes in stopping power due to implants or expanders can lead to either under-coverage of distal regions of the target or unintended dose to distal OARs [24,37]. In two different studies, the proton range through a specific type of expander was determined by testing with a sample expander, allowing for the successful treatment of patients with these expanders in place with favorable dose distributions [73,74]. Additionally, Monte Carlo methods may be able to be utilized for more accurate planning in the treatment of patients with implants or tissue expanders [75]. 

Obtaining the prior radiation treatment plan when available is a critical component of the reRT treatment planning process. Importing DICOM-RT files into the treatment planning system (TPS) allows for cumulative plan generation. By performing deformable registrations of the current proton reRT planning image set and prior simulation scan, cumulative doses to target volumes and OARs can be calculated and evaluated [56]. This allows for careful consideration of the location and amount of additional dose delivery that will optimize locoregional disease control while also informing optimal dose reduction or avoidance to critical OARs such as the heart, lungs, and brachial plexus.

### 2.6. Ongoing Trials Investigating Proton ReRT for Breast Cancer

The clinical trial registry of the National Institutes of Health (NIH) lists one clinical trial that is currently investigating reirradiation for breast cancer patients using PBT: Prospective Evaluation of Pencil Beam Scanning Proton Therapy for Previously Irradiated Tumors (The New York Proton Center NCT05313191). The breast cohort of this study includes three groups of patients: (1) patients receiving partial breast reRT via PBS PBT, (2) patients receiving regional lymph node and breast/chest wall reRT via PBS PBT, and (3) patients being treated on a breast reRT registry. The primary outcome of the study is the rate of Common Terminology Criteria for Adverse Events (CTCAE) v5.0 grade 3 or greater acute and late treatment effects within one year of the definitive reRT completion. The study is comparing the outcomes and toxicities of patients receiving PBS PBT compared to the historical outcomes of using photon reRT for breast cancer.

### 2.7. Future Directions

There are still many questions that require further exploration regarding the optimal delivery of reRT for breast cancer recurrence or new primary disease. Additional study is needed to determine the optimal total dose of reRT, as recurrences may be more resistant to radiation and other therapies, suggesting that patients with recurrences might benefit from a full dose or greater dose of reRT [17,18,76]. For example, higher reRT doses have been associated with improved locoregional control and OS in patients with recurrent non-small cell lung cancer and other disease sites [77,78]. However, excessive dose escalation may lead to severe toxicities. For example, a study attempting to determine the optimal reRT dose for recurrent esophageal squamous cell carcinoma found that a reRT dose of above 50 Gy was associated with improved OS, but doses above 60 Gy were associated with severe toxicities [79]. Studies attempting to determine the optimal reRT dose for breast cancer would be informative to the development of clinical guidelines. It is also unknown what dose fractionation scheme is optimal for tumor control and late toxicity mitigation in the reRT setting. Some studies have utilized a conventionally fractionated regimen, such as 1.8 Gy in one daily fraction, whereas others have utilized hyperfractionated regimens, such as two daily fractions of 1.5 Gy, which may be beneficial as hyperfractionated regimens allow for increased normal tissue recovery, reducing late toxicities [54,56]. In the setting of curative-intent breast cancer reRT, hypofractionation with delivery of a higher dose per fraction is often avoided due to concern for excess toxicities such as fibrosis, edema, brachial plexopathy, and neuropathy, which have been associated with higher dose per fraction regimens and would be of particular concern given the already elevated risk for severe adverse effects from the cumulative impact of multiple courses of radiotherapy [80]. Ultimately, the ideal dose-fractionation regimen in the setting of breast reRT requires further study to understand the optimal approach to attain maximal disease control while minimizing treatment-related toxicities. Considerable care in total dose selection is necessary and should be guided by BED estimations, available data, and clinical experience.

Additionally, a better understanding of the incidence and nature of adverse effects of reRT on tissues is needed from longer-term follow-up of patients receiving reRT for breast cancer. Further exploration is also warranted into the impact of the interval between RT treatment courses on disease outcomes and severity of toxicities, although the limited data currently available for breast cancer suggests that shorter intervals may lead to increased risks of severe toxicities [15]. For CNS tumor radiation treatment, some normal tissue recovery has been observed in small animal models over time, and a time-dependent model on tissue recovery after initial radiation has been developed and can be used to determine a safe reRT dose-fractionation schedule [62,81]. The development of a similar model for breast cancer would be beneficial for clinicians to develop optimal reRT plans. Investigation into the optimal treatment fields/volumes to be included (local only versus comprehensive nodal basins, etc.) for different recurrent or primary disease presentations would also be beneficial to the development of reRT clinical guidelines. For example, historically, physicians have been hesitant to recommend reRT for IMN recurrences due to concerns for toxicities to the heart or the perception that this recurrence presentation is incurable; however, data suggest that patients with isolated IMN recurrence benefit from reRT to this region [32]. Additionally, for patients with skin involvement or gross disease at the time of reRT, hyperthermia has been provided in conjunction with proton reRT as a radiosensitizer, although this may lead to increased rates of severe toxicities; thus, further study into optimal handling of these clinical scenarios is also warranted [52].

The efficacy and potential toxicities of combining PBT reRT with immunotherapies and other systemic therapies are also unknown but important, given that two of the PBT reRT studies concluded that distant recurrence is a common mode of disease control failure [54,57]. Systemic therapies have the potential to enhance the effects of radiation, which may result in improved local disease control, but this may also lead to more severe side effects [82]. Indeed, a randomized trial in head and neck carcinoma found that salvage surgery followed by chemotherapy and photon reRT resulted in improved disease-free survival compared to salvage surgery alone but that the toxicity rates were high [83]. However, a phase II trial of PBT with concurrent chemotherapy for non-resectable non-small-cell lung cancer found that this option was efficacious, and rates of toxicities were acceptable [84]. Chemotherapy provided before PBT can lead to reduced target volumes, which in one study led to reduced toxicities without a compromise in disease control [85]. Radiation, particularly PBT, given its higher RBE, may also have an immunostimulatory effect, potentially increasing the effect of immunotherapies. One mechanism by which this occurs is through the induction of targeted cells to release tumor antigens and disease-associated molecular patterns (DAMPs), allowing for the increased activation of antigen-presenting cells, which then leads to increased activation of lymphocytes. Some preclinical studies have suggested that proton therapy may be more immunogenic than photons, possibly due to higher RBE and LET [86]. Radiation can also have immunosuppressive effects, reducing the efficacy of immunotherapy treatments [87]. For example, immune cells are sensitive to radiation and can be killed at much lower doses than tumor cells, and radiation can also cause the upregulation and accumulation of immunosuppressive cells such as regulatory T cells, myeloid-derived suppressor cells, and M2-phenotype tumor-associated macrophages. The increased precision of dose delivery afforded by PBT compared to photons minimizes dose to nearby normal tissue, which might help reduce immunosuppressive components of the body’s response to radiation therapy [86]. Overall, further study is warranted to develop a better understanding of the safety and efficacy of combining PBT reRT with systemic therapies to further improve disease control outcomes in the recurrent setting. 

Additionally, the cost-effectiveness of PBT has been called into question, given that it is currently a significantly more expensive treatment compared with photon therapy. Although there are not currently any studies on the cost-effectiveness of PBT in the breast reRT setting, one study did find PBT for breast cancer to be cost-effective for women with one or more cardiac risk factors starting at a mean heart dose (MHD) of 5 Gy and cost-effective for women with no cardiac risk factors starting at a MHD of 9 Gy [88]. Given that cumulative doses to OARs, including the heart, will be increased in the reRT setting, it can be speculated that PBT for breast reRT will be cost-effective in many cases by helping to prevent toxicities such as heart disease, but further study into this topic is warranted.

## 3. Conclusions

PBT offers dosimetric advantages compared to photon therapy in breast cancer treatment due to its differential dose deposition characteristics and lack of exit dose. This inherent characteristic of the proton beam is particularly important in the setting of reRT, as OARs previously exposed to RT are at risk of repeat exposure and the potential heightened adverse impact of additional RT dose. Breast tumor recurrence or new primary breast cancers are often treated with mastectomy or, in the setting of early-stage low-risk in-breast recurrence, salvage breast-conserving surgery followed by PBI or brachytherapy. Patients with high-risk disease features, however, may benefit from higher doses and more comprehensive RT. Studies to date have shown similar effectiveness of reRT utilizing PBT compared to photons, but as these largely reported relatively short-term follow-up endpoints, continued reporting of clinical experiences with longer-term outcomes and toxicities are needed to understand the full scope of early and late effects of reRT. Given the superior dosimetric profile of proton reRT and the current outcomes reported, proton reRT for high-risk breast cancer recurrence or new primary is an attractive option and may be the most optimal external beam radiotherapy option for these patients. Proton reRT is also an attractive option for patients who are medically inoperable, as radiation would be their sole opportunity for local disease control. Further study is needed to address remaining knowledge gaps and determine the ideal approach for the delivery of reRT in the setting of high-risk breast cancer recurrence or new primary tumors.

## Figures and Tables

**Table 1:** Summary of studies investigating proton reirradiation (reRT) for breast cancer.

Study	Study Type	Population	Key Results
Proton Collaborative Group (PCG) Thorpe et al. (2019) [51]	Prospective	50 patients receiving proton reRT (24% PBS-PBT, 52% US-PBT, 24% unspecified)	12-month LRFS: 93.0% 12-month OS: 97.0% Acute grade 3 toxicities: 10.0% Late grade 3 toxicities: 8.0%
Washington University School of Medicine Gabani et al. (2019) [52]	Retrospective	16 patients receiving PS-PBT reRT	18-month LRFS: 100% 18-month DMFS: 93.3% 18-month OS: 88.9% Acute grade 3–4 skin toxicities: 5 patients (31.2%) Late grade 3–4 fibrosis: 3 patients (18.8%)
Mayo Clinic Fattahi et al. (2020) [15]	Retrospective	72 patients undergoing reRT, 20 (28%) of whom received IMPT	2-year LRFS: 74.6% 2-year OS: 65.6% Grade 3 adverse events: 13%
University of Pennsylvania LaRiviere et al. (2020) [54]	Retrospective	27 patients receiving proton reRT (29.0% DS-PBT, 66.7% PBS-PBT, 3.7% combined DS-PBT and PBS-PBT)	12-month LRFS: 78.5% 12-month RFS: 62.4% 12-month OS: 78.5% Acute grade 3 radiation dermatitis: 2 patients (7.4%) Acute grade 3 breast pain: 2 patients (7.4%) Late grade 3 dermatitis: 1 patient (3.7%) Late grade 3 breast pain: 1 patient (3.7%) Late grade 4 dermatitis: 1 patient (3.7%)
New York Proton Center/Memorial Sloan Kettering Cancer Center Choi et al. (2021) [56]	Retrospective	46 patients receiving proton reRT (70% US-PBT, 30% PBS-PBT)	2-year DMFS: 92.0% 3-year DMFS: 60.0% 2-year OS: 93.6% 3-year OS: 88.1% Acute grade 3 events: 30.4% Late grade 3 events: 8.7%
Rutgers Cancer Institute of New Jersey Sayan et al. (2022) [57]	Retrospective	15 patients undergoing reRT, 12 (80%) of which received PBT (unspecified type)	Locoregional recurrence: 27% Distant metastasis after 14 months: 33% Any grade 3 toxicity: 13%

PBS-PBT = pencil beam scanning proton therapy; US-PBT = uniform scanning proton therapy; PS-PBT = passive scattering proton therapy; DS-PBT = double scattering proton therapy; LRFS = local recurrence-free survival; OS = overall survival; DMFS = distant metastasis-free survival; RFS = recurrence-free survival.
